# The efficacy of Sijunzi on immune function in patients with gastrointestinal cancers after surgery: Integrating systematic review and network pharmacology

**DOI:** 10.1097/MD.0000000000041419

**Published:** 2025-02-07

**Authors:** Zhulin Wu, Weiqing Zhang, Wangdong Miao, Siyi Li, Jing Xie, Wensong Lu, Yuting Yan, Lisheng Peng, Weijun Luo

**Affiliations:** a Department of TCM, People’s Hospital of Longhua, Shenzhen, Guangdong, China; b Department of Hepatology, The Fourth Clinical Medical College of Guangzhou University of Chinese Medicine, Shenzhen, Guangdong, China.

**Keywords:** compound decoction, gastrointestinal cancers, immune, randomized controlled trials, TCM

## Abstract

**Background:**

Patients with gastrointestinal cancer often have impaired immune function after surgery. This study aimed to assess the efficacy of Sijunzi decoction (SJZD) on immune function in patients with gastrointestinal cancers after surgery.

**Methods:**

The electronic databases, including CNKI, Wanfang, SinoMed, Weipu, PubMed, Web of Science, EMBASE, and Cochrane databases were retrieved (March 1, 2024), and the randomized controlled trials (RCTs) that met the criteria were included. Methodologic quality assessment of RCTs was performed using the Cochrane risk of bias tool. The data of RCTs were acquired and analyzed by meta-analysis by Review Manager 5.3, and the quality of the evidence followed the Grading of Recommendations, Assessment, Development and Evaluations approach. This research was registered in the International Platform of Registered Systematic Review and Meta-analysis, 202440001. Based on the network pharmacology, relationships between key genes of SJZD and tumor-infiltrating immune cells in gastrointestinal cancers were explored.

**Results:**

Thirteen articles (RCTs) were included, containing 1010 patients with gastrointestinal cancers after surgery. The results of meta-analysis revealed that SJZD with conventional therapies could improve CD3^+^ T lymphocyte (mean difference [MD] = 5.73, 95% confidence interval [CI]: 2.07–9.39, *P* = .002), CD4^+^ T lymphocyte (MD = 5.86, 95% CI: 3.90–7.82, *P* < .00001), CD4^+^/CD8^+^ (MD = 0.29, 95% CI: 0.15–0.43, *P* < .0001), and reduce CD8^+^ T lymphocyte (MD = −2.44, 95% CI: −4.03 to −0.85, *P* = .003) compared with conventional therapies alone. In addition, the funnel plot showed the included RCTs might have publication bias. The Grading of Recommendations, Assessment, Development and Evaluations classification showed low-quality evidence for CD4^+^, CD8^+^, and CD4^+^/CD8^+^, and very low-quality evidence for other indicators. The network pharmacology results suggested that SJZD may exert effects by regulating the immune cells in the microenvironment of gastrointestinal cancers.

**Conclusion:**

SJZD could enhance the immune function of patients with gastrointestinal cancers after surgery. Due to the low quality of articles, more high-quality RCTs are needed to improve the level of evidence.

## 1. Introduction

Gastrointestinal cancers are a huge global health problem worldwide.^[1]^ Gastrointestinal cancers mainly consist of esophageal cancer, gastric cancer, and colorectal cancer, and previous research showed that gastrointestinal cancers are commonly identified as the leading causes of cancer deaths.^[2]^ Surgery is an important treatment method for gastrointestinal cancers, but patients often have impaired immune function, difficulty in eating, and weakened anabolism after gastrointestinal surgery.^[3]^ With these inadequacies, there is a great need for adjunct treatments, and the decreased immune function also needs to be solved urgently.

In China, traditional Chinese medicine (TCM) has a long history of more than 2000 years in the treatment of various diseases,^[4]^ playing an important role in the healthcare system of China. TCM could modulate the composition of gut microbiota and directly improve the immune system.^[5]^ Preceding studies indicated that Sijunzi decoction (SJZD), which was derived from the classic herb monograph “Tai Ping Hui Min He Ji Ju Fang” written in the Song dynasty, could improve the immune function (such as CD3^+^ and CD4^+^ T lymphocytes) of patients with gastrointestinal cancers.^[6]^ SJZD is a Chinese classical formula for “health-strengthening” (Fuzheng or Jianpi in Chinese), which is generally used in the treatment of gastrointestinal cancers in China. Nevertheless, SJZD is still controversial in improving immune function.

A randomized clinical trial (RCT) has shown that SJZD combined with adjuvant chemotherapy could improve CD4^+^/CD8^+^ in comparison with adjuvant chemotherapy alone,^[7]^ but another RCT reported that there was no statistical difference in CD4^+^/CD8^+^ between the treatment group and the control group.^[8]^ Moreover, the associations between SJZD and immune cells still need to be explored. Thus, systematic review/meta-analysis and network pharmacology were utilized to explore the efficacy of SJZD on immune function in patients with gastrointestinal cancers after surgery, in order to provide a scientific basis for the application of SJZD.

## 2. Methods

### 2.1. A systematic review and meta-analysis

#### 2.1.1. Inclusion criteria

##### 2.1.1.1. Type of articles

The analysis was limited to RCTs, with or without blinding. This study was registered in the International Platform of Registered Systematic Review and Meta-analysis Protocols,^[9]^ 202440001. The meta-analysis does not require an ethical statement.

##### 2.1.1.2. Participants

All adults who met the diagnostic criteria of gastrointestinal cancers, including gastric cancer, colorectal cancer, and esophageal cancer, were enrolled. In these RCTs, patients with gastrointestinal cancers should be treated surgically, and the diagnosis was confirmed by pathological examinations.

##### 2.1.1.3. Intervention and control

The treatment groups were treated with nasogastric feeding or oral administration of SJZD plus conventional therapies after operation, SJZD must be composed of Radix Codonopsis (or Radix Ginseng), Rhizoma Atractylodis Macrocephalae, Poria, and Radix Glycyrrhizae Preparata, and cannot be modified to ensure homogeneity. The control groups were treated with conventional therapies after surgery, including nutrition support or adjuvant chemotherapy.

##### 2.1.1.4. Observation indicators

Observation indicators were as follows: included articles had to report on immune cells, including CD3^+^, CD4^+^, CD8^+^, CD4^+^/CD8^+^, natural killer (NK) cells, or immunoglobulin (IgA, IgG, and IgM), as the outcome measure; albumin (ALB); safety (adverse events) was evaluated descriptively.

#### 2.1.2. Exclusion criteria

The exclusion criteria were as follows: the treatment groups were treated with modified SJZD; the indicators did not involve immune function; intervention measures included other TCM treatments, such as acupuncture; the intervention in the control groups included TCM therapy; repetitive articles, protocol, plagiarized literature, incomplete data; articles on non-Chinese or non-English literature; case report, theoretical studies, review, and experimental articles.

#### 2.1.3. Search strategy

A computer search of English databases such as PubMed, Cochrane Library, Embase, Web of Science, and Chinese databases such as China National Knowledge Infrastructure (CNKI) database, Wanfang Data, China Biomedical Literature database, and VIP Chinese Journal Full-text (Weipu) database was performed. These databases were searched from inception until March 1, 2024. The following search terms are used: “gastrointestinal neoplasms OR Gastrointestinal Neoplasm OR Neoplasm, Gastrointestinal OR Neoplasms, Gastrointestinal OR Cancer of Gastrointestinal Tract OR Gastrointestinal Tract Cancer OR Gastrointestinal Tract Cancers OR Cancer of the Gastrointestinal Tract OR Gastrointestinal Cancer OR Cancer, Gastrointestinal OR Cancers, Gastrointestinal OR Gastrointestinal Cancers OR Stomach Neoplasms OR Esophageal Neoplasms OR Colorectal Neoplasms OR Colonic Neoplasms OR Rectal Neoplasms OR malignant tumor of small intestine OR esophageal cancer OR esophageal carcinoma OR gastric carcinoma OR gastric cancer OR colorectal carcinoma OR colorectal cancer OR Colon cancer OR colon carcinoma OR rectal carcinoma OR rectal cancer,” “traditional Chinese medicine OR Chinese materia medica,” “randomized,” and, “sijunzi OR si jun zi.”

#### 2.1.4. Literature screening, quality evaluation, and data extraction

The retrieved literature during the database search was screened by 2 authors independently in order to reduce bias. Literature that did not meet the inclusion criteria is excluded by reading titles and abstracts. Then, the quality of the literature was assessed and the extracted study data were sorted out by Excel 2013 software (Microsoft Office). The methodological quality of the RCTs in the literature was evaluated by 7 items from the Cochrane risk of bias tool (https://methods.cochrane.org/bias/resources/cochrane-risk-bias-tool), including random sequence generation, allocation concealment, participant and subject blinding, outcome assessment blinding, completeness of outcomes, outcome reporting bias, and other biases. The methodological quality was recorded as “low risk,” “high risk,” or “unclear risk.” The RCTs that meet all criteria are considered to have a lower risk of bias, while RCTs that do not meet any criteria are considered to have a higher risk of bias. Otherwise, RCTs were thought to have an unclear risk of bias.

#### 2.1.5. Statistical analysis

Meta-analysis was utilized to merge the data of the included RCTs by using Review Manager 5.3 (London, UK). In this research, all indicators are continuous variables, and the continuous variables used the mean difference (MD) as the effect size measure. The results were calculated with 95% confidence intervals (95% CIs). Moreover, heterogeneity between articles was assessed by the *I*^2^ test. When the value of *I*^2^ is <50%, the fixed-effects model was used to perform the analysis, and if the value of *I*^2^ is >50%, the random-effects model or descriptive analysis was performed. The sensitivity analysis was utilized to assess the robustness of the meta-analyses by omitting the articles one by one. The results of meta-analysis were shown by forest plot, *P* < .05 was considered significant, and a funnel plot was drawn to analyze publication bias. The Grading of Recommendations, Assessment, Development and Evaluations proGDT online tool (https://www.gradepro.org/) was used to assess the evidence quality for outcome indicators based on 5 downgrading factors: risk of bias, inconsistency, indirectness, imprecision, and potential publication bias.

### 2.2. Network pharmacology

#### 2.2.1. Screening key genes of SJZD and gastrointestinal cancers

The HERB database (http://herb.ac.cn/)^[10]^ was utilized to obtain the target genes of SJZD. Potential therapeutic target genes related to gastrointestinal cancers were collected using “gastric cancer,” “esophageal cancer,” “colorectal cancer,” and “rectum cancer” as the keywords from the MalaCards (https://www.malacards.org/). Common genes of gastrointestinal cancers and SZJD were screened by using Venny 2.1 (https://bioinfogp.cnb.csic.es/tools/venny/). Subsequently, the protein–protein interaction (PPI) network of the common genes was analyzed by setting the default parameters from the Search Tool for the Retrieval of Interacting Genes database (https://string-db.org/).^[11]^ Based on the calculation of degree in the PPI network, key genes were screened using the CytoHubba plugin of Cytoscape 3.7.1 (https://cytoscape.org/).

#### 2.2.2. Immune infiltrates correlation analysis using TIMER

Relationships between immune infiltrates and key genes were explored by the Tumor Immune Estimation Resource tool (TIMER, https://cistrome.shinyapps.io/timer/).^[12]^ TIMER is an effective tool to display a comprehensive understanding of tumor-immune interactions, and the purity-corrected partial Spearman correlation (partial-cor) and *P* value provided by TIMER are shown in this study.

## 3. Results

### 3.1. Search results

**Figure 1.**
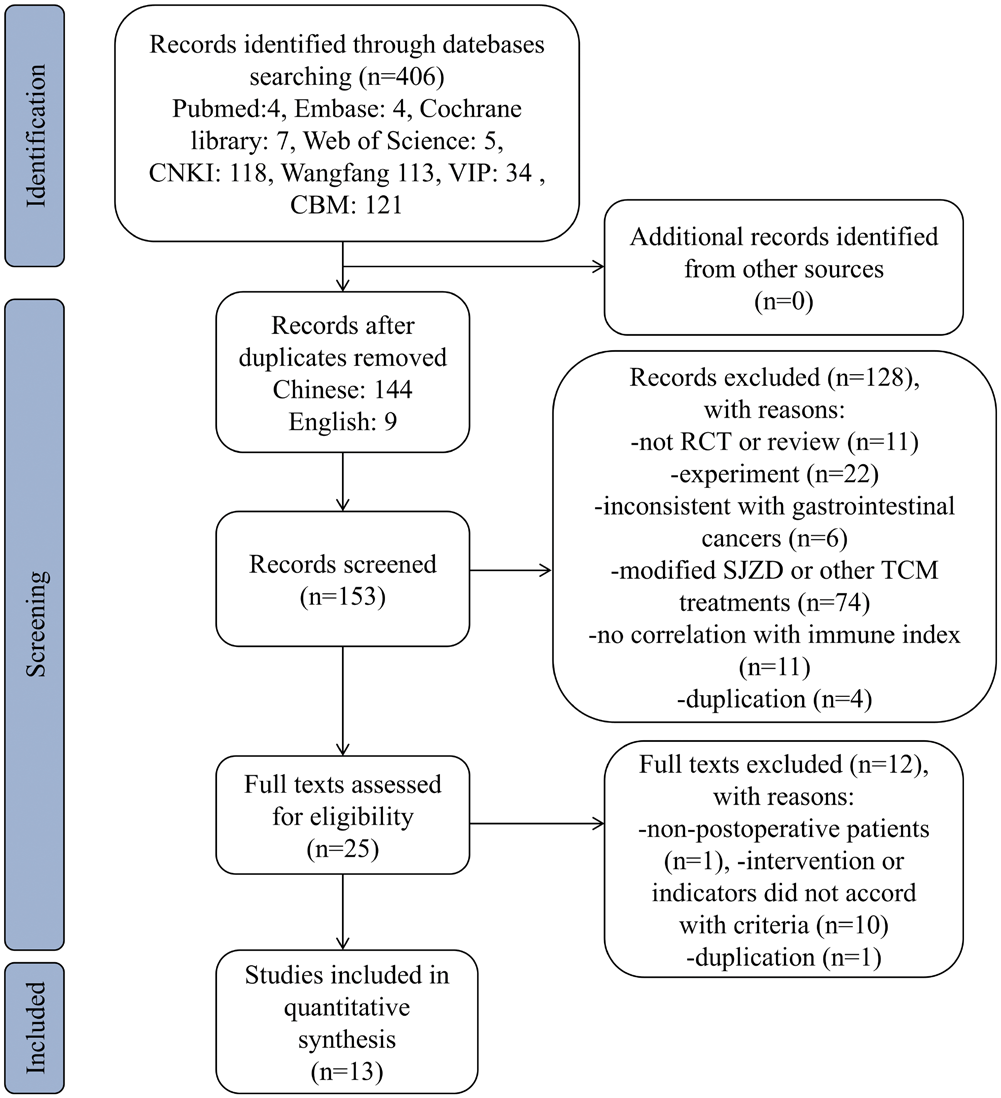
Flow chart of the study selection process. CBM = China Biomedical Literature, CNKI = China National Knowledge Infrastructure, RCT = randomized clinical trial, SJZD = Sijunzi decoction, TCM = traditional Chinese medicine.

In the Chinese databases, a total of 386 related articles were collected, including 118 from CNKI, 113 from Wangfang, 34 from VIP, and 121 from China Biomedical Literature. Through the search of the English database, 4, 5, 4, and 7 articles were found in PubMed, Web of Science, EMBASE, and Cochrane Library, respectively. According to the inclusion and exclusion criteria, 13 Chinese RCTs^[6-8,13-22]^ were selected (Fig. 1).

### 3.2. Results of methodological quality evaluation

**Figure 2.**
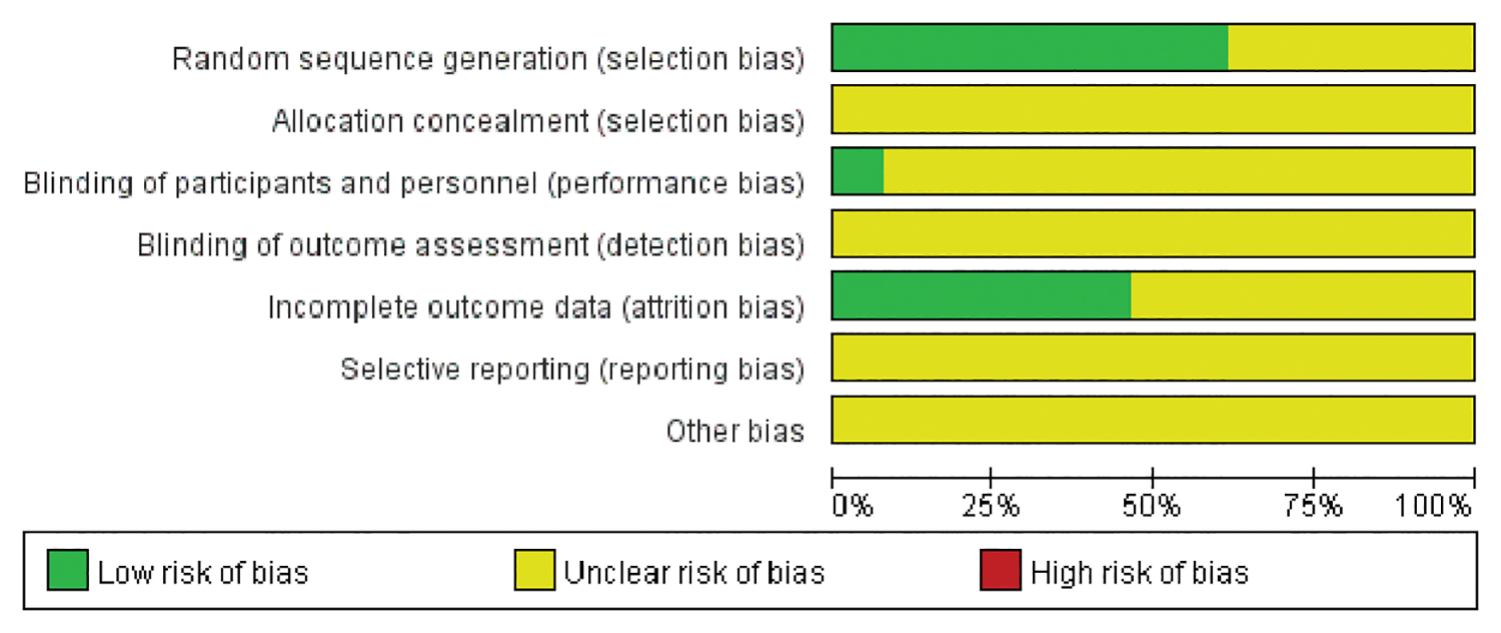
Risk of bias graph.

**Figure 3.**
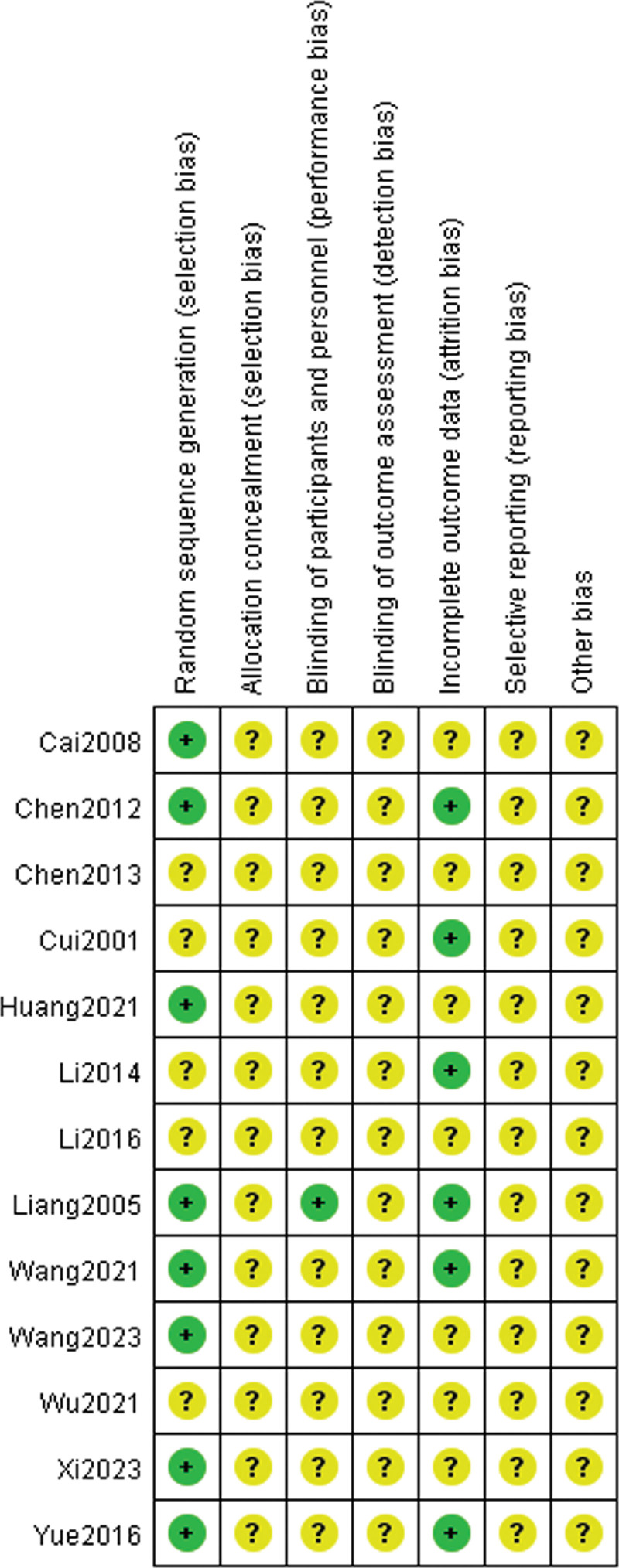
Risk of bias summary.

Eight RCTs^[6,7,14,16,17,19,20,22]^ included in this study described the specific random sequence generation method, all of which were random number table methods. Only 1 RCT mentioned the blinding of participants, other RCTs did not describe specific allocation concealment methods or blinding of participants, personnel, and outcome assessment. Six RCTs^[7,13,15,16,20,22]^ described incomplete outcome data. Other RCTs did not clearly describe the relevant items, which were considered “unclear.” The risk of bias graph and risk of bias summary are displayed in Figures 2 and 3.

### 3.3. The basic characteristics of the 13 RCTs

Except for 1 RCT that did not mention the baseline characteristics,^[13]^ the other included RCTs mentioned that there was no significant difference in baseline characteristics (gender, age, and pathological type) between the experimental group and the control group. All patients with gastrointestinal cancer in the experimental group were treated with SJZD and conventional therapies after surgery. The control group was treated with conventional therapies after surgery. In this study, a total of 13^[6-8,13-22]^ articles were selected, including 1010 patients with gastrointestinal cancers, involving 2 observation indexes: immune function, and ALB; 1 article^[16]^ mentioned adverse reactions (Table 1).

**Table 1:** The basic characteristics of the 13 articles (RCTs).

Study	Sample (T/C)	Type	Intervention (T/C)	Course	Indicators
Cai et al^[6]^	18/21	Gastric cancer	SJZD + nutrition support/nutrition support	9 d	①
Chen^[22]^	19/20	Esophageal cancer	SJZD + nutrition support/nutrition support	7 d	①②
Chen^[21]^	22/22	Gastric cancer	SJZD + nutrition support/nutrition support	7 d	①②
Cui and Jin^[13]^	16/16	Esophageal cancer	SJZD + adjuvant chemotherapy/adjuvant chemotherapy	3 wk	①
Huang and Wang^[14]^	51/51	Gastric cancer	SJZD + nutrition support/nutrition support	2 wk	①②
Li^[15]^	30/30	Esophageal cancer	SJZD + adjuvant chemotherapy/adjuvant chemotherapy	4 wk	①
Li et al^[8]^	69/69	Colorectal cancer	SJZD + nutrition support/nutrition support	8 d	①
Liang et al^[16]^	47/46	Gastrointestinal cancers	SJZD + nutrition support/nutrition support	7 d	①
Wang et al^[20]^	41/41	Colorectal cancer	SJZD + nutrition support/nutrition support	7 d	①
Wang and Li^[19]^	42/42	Gastric cancer	SJZD + nutrition support/nutrition support	7 d	①②
Wu et al^[18]^	44/43	Gastric cancer	SJZD + nutrition support/nutrition support	7 d	①②
Xi and Xao^[17]^	40/40	Gastric cancer	SJZD + nutrition support/nutrition support	7 d	①②
Yue^[7]^	65/65	Rectal cancer	SJZD + adjuvant chemotherapy/adjuvant chemotherapy	8 wk	①

Observation indicators: ①immune cells (CD3+, CD4+, CD8+, CD4+/CD8+, and NK), immunoglobulin (IgA, IgM, and IgG); ②ALB.

ALB = albumin, AR = adverse reactions, C = control, Ig = immunoglobulin, NA = not applicable, NK = natural killer, RCT = randomized clinical trial, SJZD = Sijunzi decoction, T = treatment.

### 3.4. Results of meta-analysis

#### 3.4.1. Meta-analysis of SJZD in regulating immune function

**Figure 4.**
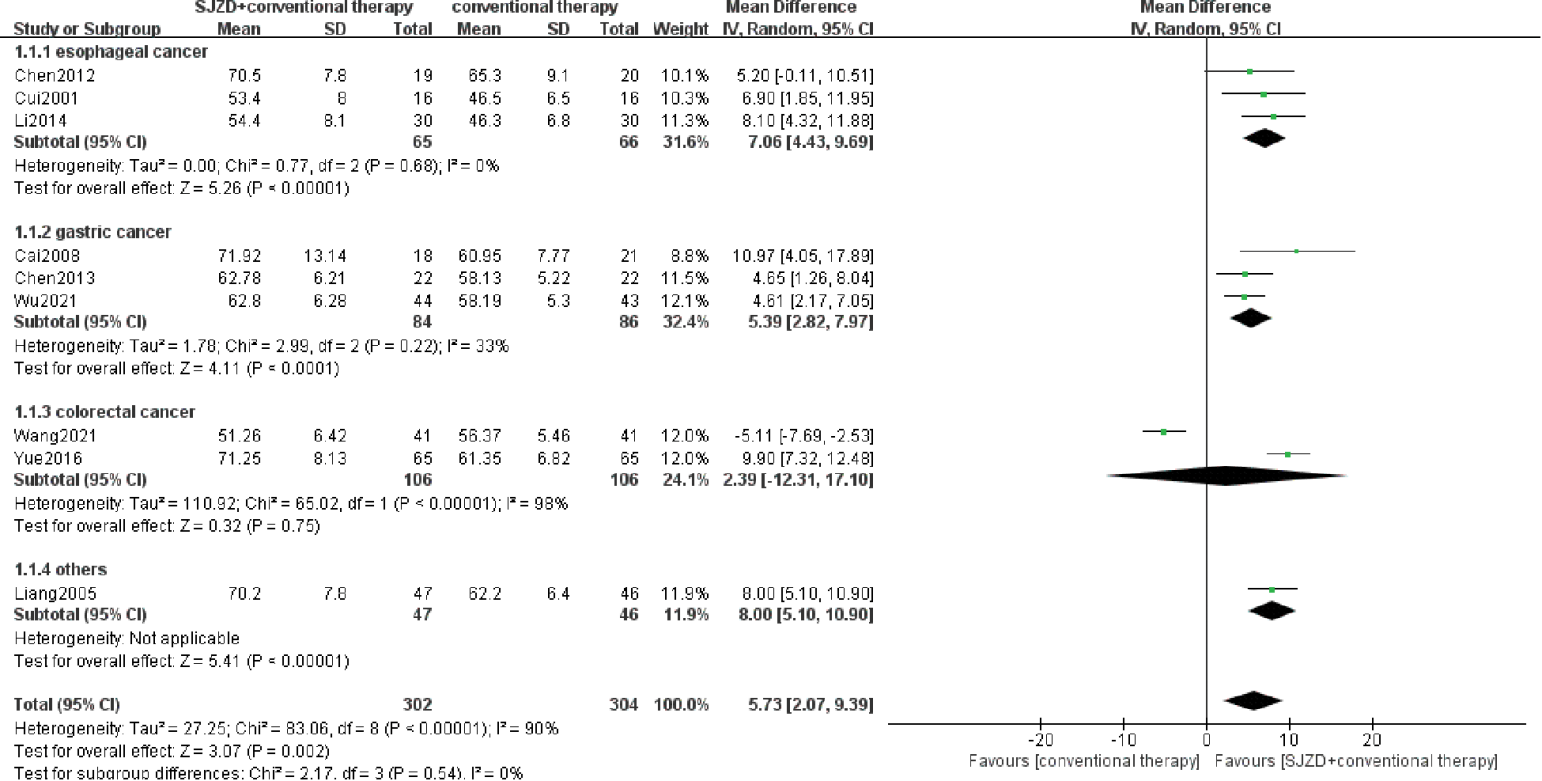
Meta-analysis of CD3^+^ T lymphocyte. CI = confidence interval, IV = inverse variance, SD = standard deviation, SJZD = Sijunzi decoction.

**Figure 5.**
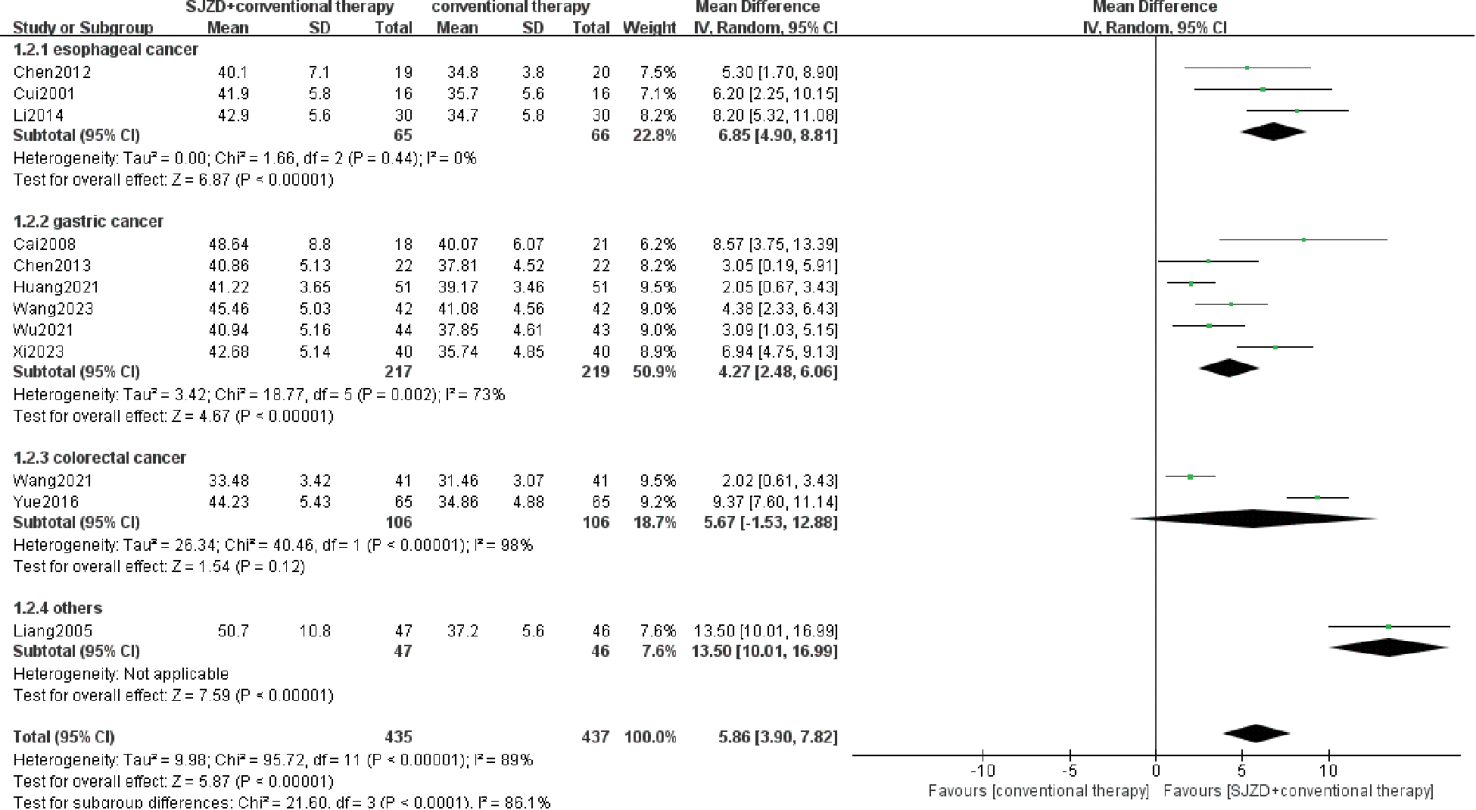
Meta-analysis of CD4^+^ T lymphocyte. CI = confidence interval, IV = inverse variance, SD = standard deviation, SJZD = Sijunzi decoction.

**Figure 6.**
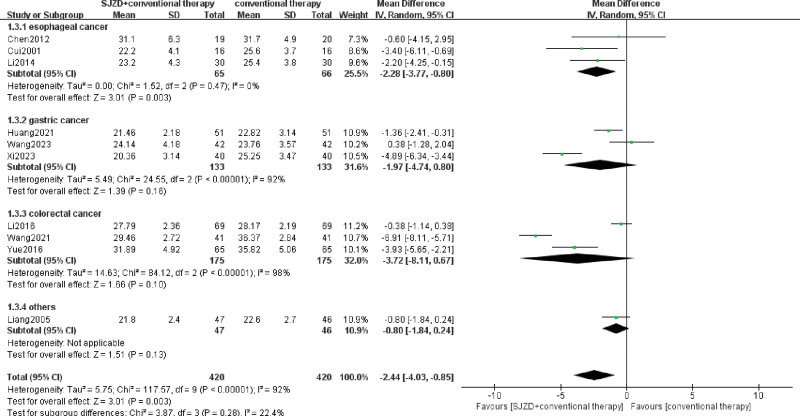
Meta-analysis of CD8^+^ T lymphocyte. CI = confidence interval, IV = inverse variance, SD = standard deviation, SJZD = Sijunzi decoction.

**Figure 7.**
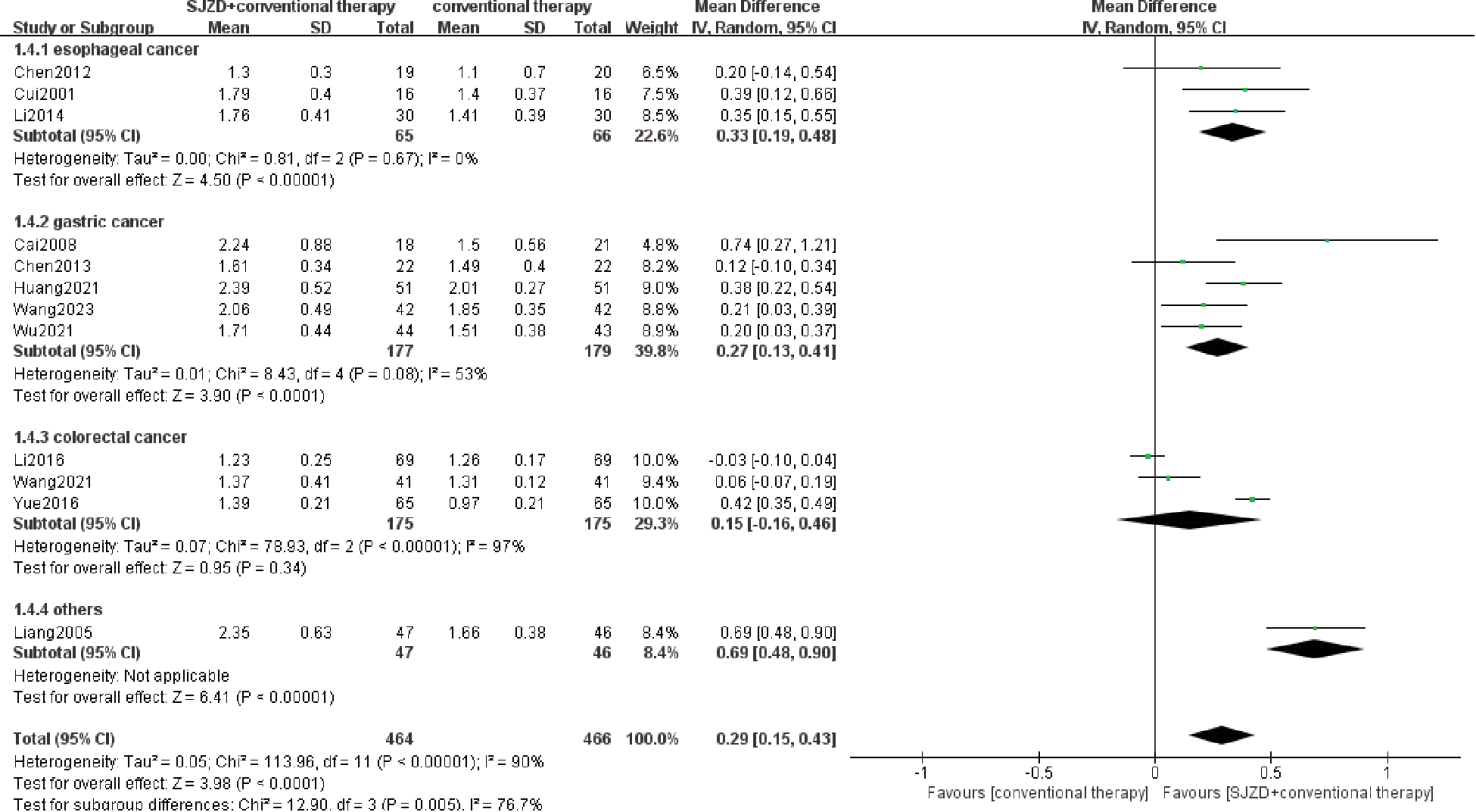
Meta-analysis of CD4^+^/CD8^+^. CI = confidence interval, IV = inverse variance, SD = standard deviation, SJZD = Sijunzi decoction.

The main indicators involving the immune function index, including CD3^+^, CD4^+^, CD8^+^, CD4^+^/CD8^+^, NK, IgA, IgM, and IgG, were assessed, and 13 RCTs^[6-8,13-22]^ were included. Heterogeneity among immune cells was high, with *I*^2^ >50%, and the random effect model was used for meta-analysis. The results were demonstrated in the forest plots (Figs. 4–7), indicating that SJZD combined with conventional therapies after operation could increase CD3^+^ (MD = 5.73, 95% CI = 2.07–9.39, *P* = .002), CD4^+^ (MD = 5.86, 95% CI = 3.90–7.82, *P* < .00001), and CD4^+^/CD8^+^ (MD = 0.29, 95% CI = 0.15–0.43, *p* for effect < 0.0001), and reduce CD8^+^ (MD = −2.44, 95% CI = −4.03 to −0.85, *P* = .003). Moreover, the results of subgroup analysis showed that SJZD could increase the CD3^+^, CD4^+^, and CD4^+^/CD8^+^ of patients with esophageal or gastric cancer, and decrease the CD8^+^ of esophageal cancer. In addition, SJZD may elevate the immunoglobulin (IgM, IgA, and IgG) of patients with gastrointestinal cancers after surgery, and there was no significant difference in NK cells between the 2 groups (Table 2). The sensitivity analysis revealed that the results for all indicators of immune function, with the exception of IgA, demonstrated robustness (Supplementary Tables S1–S7, Supplemental Digital Content, http://links.lww.com/MD/O333).

**Table 2:** Meta-analysis of other indexes.

Indicators	Sample (T/C)	*I*^2^/%	Model	MD	95% CI	*P*
NK^[8,14,16]^	115/115	94	Random	5.03	(−0.94 to 10.99)	.10
IgM^[6,8,17,18,21,22]^	237/239	95	Random	0.35	(0.10–0.60)	.007
IgA^[6,8,17,18,21,22]^	237/239	97	Random	0.44	(0.03–0.86)	.03
IgG^[6,8,17,21,22]^	197/199	91	random	1.95	(0.72–3.19)	.002

C = control, CI = confidence interval, Ig = immunoglobulin, MD = mean difference, NK = natural killer, T = treatment.

#### 3.4.2. Meta-analysis of ALB

**Figure 8.**
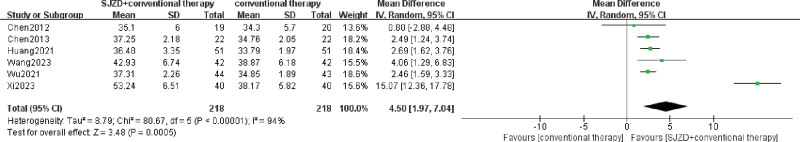
Meta-analysis of albumin. CI = confidence interval, IV = inverse variance, SD = standard deviation, SJZD = Sijunzi decoction.

Five articles (RCTs)^[14,17-19,21,22]^ reported the ALB index. The *I*^2^ of the heterogeneity test is 94% (*P* < .00001), suggesting high heterogeneity and the random effect model was used for meta-analysis. The result of meta-analysis showed that SJZD combined with conventional therapies could increase ALB (MD = 4.50, 95% CI = 1.97–7.04, *P* = .0005), whose effect was superior to conventional therapies alone for gastrointestinal cancers (Fig. 8). Sensitivity analysis confirmed the robustness of result of ALB (Supplementary Table S8, Supplemental Digital Content, http://links.lww.com/MD/O333).

#### 3.4.3. Analyses for publication bias, safety evaluation, and quality of the evidence

**Figure 9.**
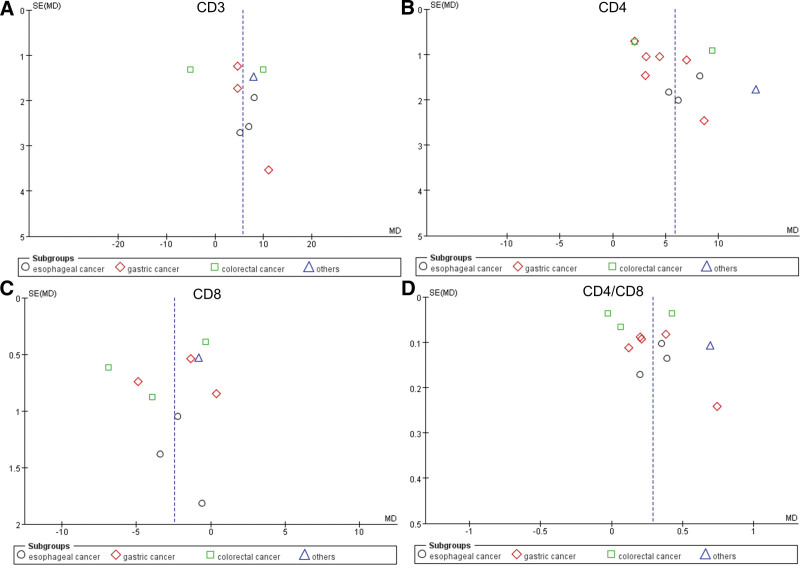
Funnel plot analysis for T lymphocytes. (A) Funnel plot of CD3^+^ cells. (B) Funnel plot of CD4^+^ cells. (C) Funnel plot of CD8^+^. (D) Funnel plot of CD4^+^/CD8^+^. MD = mean difference, SE = standard error.

In immune function (T lymphocytes), a publication bias analysis was performed by using funnel plots. As shown in Figure 9, it was found that the distribution of the funnel plot was asymmetrical. Furthermore, 1 article (RCT)^[16]^ reported adverse reactions of interventions, and no severe adverse reactions were observed in patients with gastrointestinal cancer who were in the treatment group. All outcomes were included in a Grading of Recommendations, Assessment, Development and Evaluations evidence profile table (Supplementary Figure S1, Supplemental Digital Content, http://links.lww.com/MD/O333). The evidence on CD4^+^, CD8^+^, and CD4^+^/CD8^+^ was low quality, and the evidence on other indicators was very low quality.

### 3.5. Results of network pharmacology

#### 3.5.1. Common genes and key genes of PPI networks

**Figure 10.**
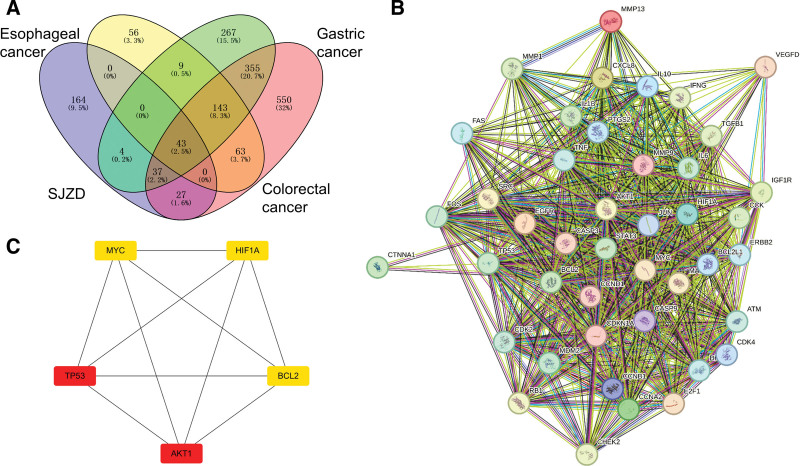
Common genes and key genes. (A) Venn diagram of common genes of SJZD and gastrointestinal cancers. (B) The PPI network of common genes. (C) The network of key genes. PPI = protein–protein interaction, SJZD = Sijunzi decoction.

A total of 275 genes of SJZD were found using HERB databases, and 1546 potential therapeutic genes for gastrointestinal cancers were obtained. After excluding the duplicate genes, a total of 43 common genes of gastrointestinal cancers and SJZD were screened (Fig. 10A).

The PPI network of common genes was established through Search Tool for the Retrieval of Interacting Genes, and there were 43 nodes and 714 edges in the interaction network (Fig. 10B). According to the calculation of degree in the PPI network, the top 5 key genes were screened, and *AKT1*, *TP53*, *MYC*, hypoxia-inducible factor 1 subunit alpha (*HIF1A*), and *BCL2* could be the potential genes for SJZD in treating gastrointestinal cancers (Fig. 10C).

#### 3.5.2. Tumor-infiltrating immune cells related to key genes

In TIMER, the data on genes come from the Cancer Genome Atlas program. Potential immunological correlations between key genes and tumor-infiltrating immune (including B cell, CD8^+^ T cell, CD4^+^ T cell, macrophage, neutrophil, and dendritic cell) were analyzed. The expression of *HIF1A* was negatively correlated with tumor purity (*P* < .05), suggesting that *HIF1A* was highly expressed in the gastrointestinal cancers microenvironment. Also, there is a correlation between *HIF1A* and immune cells (B cell, CD8^+^ cell, CD4^+^ cell, macrophage, neutrophil, or dendritic cell) in esophageal carcinoma, stomach adenocarcinoma, colon adenocarcinoma, and rectum adenocarcinoma. The result of tumor purity and tumor-infiltrating immune cells are demonstrated in Table 3.

**Table 3:** Associations between key genes and tumor-infiltrating immune cells in gastrointestinal cancers (partial Spearman correlation).

Cancer type	Genes	Purity	B cell	CD8^+^	CD4^+^	Macrophage	Neutrophil	DC
Esophageal carcinoma	*AKT1*	0.155*	0.003	−0.242*	0.082	0.047	−0.252*	−0.061
	*TP53*	0.156*	−0.1	−0.144	0.093	−0.013	−0.166*	0.064
	*MYC*	0.065	−0.123	0.015	−0.125	−0.227*	−0.098	0.088
	*HIF1A*	−0.256*	−0.164*	0.051	−0.195*	0.117	−0.063	0.168*
	*BCL2*	−0.054	0.35*	−0.05	0.256*	0.297*	0.117	0.057
Stomach adenocarcinoma	*AKT1*	0.046	−0.001	−0.126*	0.104*	−0.083	−0.137*	−0.088
	*TP53*	0.053	−0.115*	0.106*	0.013	−0.078	0.114*	0.099
	*MYC*	0.04	−0.066	−0.091	−0.086	−0.177	−0.079	−0.13*
	*HIF1A*	−0.139*	−0.139*	−0.087	0.15*	0.045	0.154	0.299*
	*BCL2*	−0.109*	0.189*	0.319*	0.57*	0.435*	0.314*	0.453*
Colon adenocarcinoma	*AKT1*	0.147*	−0.115*	0	0.34*	0.168*	0.22*	0.283*
	*TP53*	0.045	0.013	0.005	−0.052	−0.064	−0.046	0.02
	*MYC*	0.108*	−0.084	0.073	0.197*	0.109*	0.074	0.055
	*HIF1A*	−0.341*	0.307*	0.582*	0.308*	0.487*	0.658*	0.608*
	*BCL2*	−0.285*	0.353*	0.402*	0.468*	0.36*	0.41*	0.525*
Rectum adenocarcinoma	*AKT1*	−0.126	0.023	−0.081	0.241*	0.104	0.003	0.255*
	*TP53*	−0.023	0.105	−0.046	−0.061	−0.036	−0.094	−0.127
	*MYC*	−0.011	0.116	−0.068	0.153	−0.055	−0.101	0.063
	*HIF1A*	−0.383*	0.256*	0.585*	−0.037	0..238*	0.565*	0.421
	*BCL2*	−0.373*	0.441*	0.287*	0.371*	0.209*	0.078	0.359*

DC = dendritic cell

* *P* < .05.

## 4. Discussion

Surgical resection is the primary treatment for patients with gastrointestinal cancers,^[23]^ but there are still some difficulties, such as weakened anabolism and decreased immune function. Postoperative enteral nutrition support for patients with gastrointestinal cancers has a certain effect, which can improve immune function and malnutrition, and postoperative adjuvant chemotherapy can reduce the risk of cancer recurrence. However, enteral nutrition or adjuvant chemotherapy also has limitations in improving immunity. Therefore, better adjuvant treatment for gastrointestinal cancers after surgery is critical for patients’ prognosis.

TCM has been widely used as a complementary and alternative treatment throughout Chinese history.^[24]^ As a classical and important prescription of TCM, SJZD adjuvant therapy for gastrointestinal cancers could improve immune function. For instance, several studies have revealed that SJZD combined with conventional therapies can improve the CD3^+^ and CD4^+^ T lymphocytes of patients with gastrointestinal cancers.^[6,16]^ Nevertheless, there is a lack of evidence-based medicine. To establish the evidence of SJZD in treating patients with gastrointestinal cancers after surgery, 13 articles (RCTs) that met the inclusion criteria were analyzed with meta-analyses in the study. The results of meta-analysis showed that SJZD plus conventional therapies could further increase CD3^+^ T lymphocyte, CD4^+^ T lymphocyte, CD4^+^/CD8^+^, immunoglobulin (IgA, IgG, and IgM), and reduce CD8^+^ T lymphocyte of patients with gastrointestinal cancers, compared with conventional therapies alone. Thus, immune function could be improved by SJZD. The meta-analysis result also demonstrated that the SJZD could increase the value of ALB, which can reflect the nutritional status of patients. There was high heterogeneity in the meta-analysis results of immune function, which may be related to the differences in the baseline values of included RCTs. Moreover, the pooled MD was stable and the significance of the pooled MD did not change when a single RCT was removed. In addition, 1 research suggests that the side effects of adding SJZD were less but more studies are needed to pay attention to its safety in the future. For the main observation indicators, the funnel plots showed possible small-study or publication bias. The immune system plays a vital role in regulating tumor growth and metastasis, and peripheral blood T lymphocyte subsets have become an important index to predict prognosis.^[25]^ Preoperative LAR, which is determined as the serum lactic dehydrogenase to ALB, is reported to be a potential prognostic marker in patients with colorectal cancer.^[26]^

According to TCM theory, the “spleen” (pi in Chinese) is the source of the acquired constitution and the source of “vital qi” (Zhengqi in Chinese). Postoperative patients with gastrointestinal cancers often have symptoms of “spleen” insufficiency and “qi-blood” deficiency, resulting in malnutrition and decreased immune function. SJZD is composed of Radix Codonopsis (or Radix Ginseng), Rhizoma Atractylodis Macrocephalae, Poria, and Radix Glycyrrhizae Preparata, and the whole formula has the effect of invigorating the spleen and replenishing qi. The active ingredients of SJZD could regulate the secretion of gastrointestinal hormones in “spleen” deficiency syndrome rats and improve intestinal immunity by increasing the expression of T lymphocyte cells.^[27]^ Polysaccharides from Rhizoma Atractylodis Macrocephalae could significantly activate macrophages on the basis of the increased releases of cytokines (Interleukin 6, Interleukin 10, and Tumor necrosis factor-α).^[28]^ Poria is traditionally used in combination with other Chinese herbs to promote immunity, and Poria extract could enhance innate immunity by activating NK cells and promote IFN-γ secretion by type 1 T-helper cell’s immune response.^[29]^ An animal experimental study showed that Radix Codonopsis polysaccharide pretreatment maintained the immune balance of CD4/CD8 T cells.^[30]^

The result of network pharmacology demonstrated that SJZD might exert an antigastrointestinal cancer effect by regulating multiple tumor-related genes (e.g., *AKT1*, *TP53*, *MYC*, and *BCL2*). Several key genes are associated with tumor-infiltrating immune cells in different gastrointestinal cancers, indicating that the SJZD may play an antitumor effect by regulating immune cells in the tumor microenvironment. In particular, only 1 key gene (*HIF1A*) was found to be associated with tumor purity and tumor-infiltrating immune cells in all 4 types of gastrointestinal cancers. It was reported that upregulation of *HIF1A* was associated with increased immune signature, aggressive phenotypes, and worse survival rates in different cancers, and *HIF1A* expression showed a stronger correlation with immune-inhibiting signatures than with immune-promoting signatures.^[31]^ However, the specific role of *HIF1A* in different cancer types remains to be further studied.

Nevertheless, several limitations are inevitable in this research: despite our efforts, some literature was excluded from the meta-analysis due to the lack of detailed immune-related data. Significant heterogeneity can be observed in the meta-analysis, and this high heterogeneity may result from the stage and the type of gastrointestinal cancers. Thus, more large-scale clinical trial data are needed to identify the source of the heterogeneity. The results of network pharmacology reported in this study should be further verified through experimental studies.

To sum up, although high heterogeneity and asymmetrical funnel plot showed potential bias underlying the result, meta-analysis results suggested that SJZD with conventional therapies could improve immune function and ALB of patients with gastrointestinal cancer. SJZD may play a therapeutic role in gastrointestinal cancers through immune-related genes. In the future, high-quality and large-sample RCTs are needed to support our results.

## Author contributions

**Conceptualization:** Zhulin Wu, Weijun Luo.

**Data curation:** Zhulin Wu, Weiqing Zhang, Siyi Li, Jing Xie, Wensong Lu, Yuting Yan.

**Formal analysis:** Zhulin Wu, Weiqing Zhang, Siyi Li, Jing Xie, Yuting Yan, Weijun Luo.

**Software:** Zhulin Wu, Wangdong Miao, Wensong Lu, Yuting Yan, Lisheng Peng.

**Writing – original draft:** Zhulin Wu.

**Methodology:** Weiqing Zhang, Wangdong Miao, Siyi Li, Jing Xie, Yuting Yan.

**Validation:** Weiqing Zhang, Jing Xie, Lisheng Peng.

**Investigation:** Wangdong Miao, Weijun Luo.

**Visualization:** Wangdong Miao, Lisheng Peng.

**Writing – review & editing:** Siyi Li, Lisheng Peng, Weijun Luo.

**Supervision:** Wensong Lu, Lisheng Peng, Weijun Luo.

**Funding acquisition:** Weijun Luo.

## Supplementary Material


