# Supplementary material for: The efficacy of Sijunzi on immune function in patients with gastrointestinal cancers after surgery: Integrating systematic review and network pharmacology
Source: Medicine (Baltimore). 2025 Feb 7;104(6):e41419. doi: 10.1097/MD.0000000000041419 (PMC11813061; doi:10.1097/MD.0000000000041419)
Supplement: Supplementary file 1 [file medi-104-e41419-s001.pdf]

Certainty assessment							№ of patients		Effect		Certainty	Importance
№ of studies	Study design	Risk of bias	Inconsistency	Indirectness	Imprecision	Other considerations	SJZD plus conventional therapies	conventional therapies	Relative (95% CI)	Absolute (95% CI)		
**CD3**												
8	randomised trials	not serious	serious[a]	not serious	serious[b]	publication bias strongly suspected[c]	383	384	-	MD **5.73 higher** (2.07 higher to 9.39 higher)	⊕◯◯◯ Very low[a,b,c]	CRITICAL
**CD4**												
12	randomised trials	not serious	serious[a]	not serious	not serious	publication bias strongly suspected[c]	435	437	-	MD **5.86 higher** (3.9 higher to 7.82 higher)	⊕⊕◯◯ Low[a,c]	CRITICAL
**CD8**												
10	randomised trials	not serious	serious[a]	not serious	not serious	publication bias strongly suspected[c]	420	420	-	MD **2.44 lower** (4.03 lower to 0.85 lower)	⊕⊕◯◯ Low[a,c]	CRITICAL
**CD4/CD8**												
12	randomised trials	not serious	serious[a]	not serious	not serious	publication bias strongly suspected[c]	484	486	-	MD **0.29 higher** (0.15 higher to 0.43 higher)	⊕⊕◯◯ Low[a,c]	CRITICAL
**NK**												
3	randomised trials	not serious	serious[a]	not serious	serious[b]	publication bias strongly suspected[c]	115	115	-	MD **5.03 higher** (0.94 lower to 10.99 higher)	⊕◯◯◯ Very low[a,b,c]	CRITICAL
**IgA**												
6	randomised trials	not serious	serious[a]	not serious	serious[b]	publication bias strongly suspected[c]	237	239	-	MD **0.44 higher** (0.03 higher to 0.86 higher)	⊕◯◯◯ Very low[a,b,c]	IMPORTANT
**IgG**												
5	randomised trials	not serious	serious[a]	not serious	serious[b]	publication bias strongly suspected[c]	197	199	-	MD **1.95 higher** (0.72 higher to 3.19 higher)	⊕◯◯◯ Very low[a,b,c]	IMPORTANT
**IgM**												
6	randomised trials	not serious	serious[a]	not serious	serious[b]	publication bias strongly suspected[c]	237	239	-	MD **0.35 higher** (0.1 higher to 0.6 higher)	⊕◯◯◯ Very low[a,b,c]	IMPORTANT
**ALB**												
6	randomised trials	not serious	serious[a]	not serious	serious[b]	publication bias strongly suspected[c]	218	218	-	MD **4.5 higher** (1.97 higher to 7.04 higher)	⊕◯◯◯ Very low[a,b,c]	IMPORTANT

CI: confidence interval; **MD:** mean difference

Explanations

a. There was great heterogeneity, and the trends of some study results were different.
b. The total number of cases is low.
c. Publication bias strongly suspected

Supplementary Figure S1

Supplementary Table 1 Sensitivity analysis of CD3

**Study (omitted)**	**MD[95%CI]**
Chen2012	5.80 [1.82, 9.78]
Cui2001	5.61 [1.62, 9.59]
Li2014	5.44 [1.41, 9.47]
Cai2008	5.23 [1.38, 9.07]
Chen2013	5.89 [1.74, 10.05]
Wu2021	5.92 [1.54, 10.30]
Wang2021	7.10 [5.37, 8.83]
Yue2016	5.15 [1.30, 9.01]
Liang2005	5.45 [1.33, 9.57]

Supplementary Table 2 Sensitivity analysis of CD4

**Study**	**MD[95%CI]**
Chen2012	5.91 [3.84, 7.99]
Cui2001	5.84 [3.78, 7.91]
Li2014	5.65 [3.61, 7.70]
Cai2008	5.68 [3.66, 7.71]
Chen2013	6.12 [4.03, 8.22]
Huang2021	6.26 [4.19, 8.34]
Wang2023	6.03 [3.86, 8.19]
Wu2021	6.15 [4.01, 8.29]
Xi2023	5.77 [3.66, 7.87]
Wang2021	6.27 [4.19, 8.34]
Yue2016	5.44 [3.61, 7.28]
Liang2005	5.20 [3.40, 7.00]

Supplementary Table 3 Sensitivity analysis of CD8

**Study**	**MD[95%CI]**
Chen2012	-2.58 [-4.25, -0.92]
Cui2001	-2.35 [-4.04, -0.66]
Li2014	-2.46 [-4.19, -0.74]
Huang2021	-2.57 [-4.41, -0.73]
Wang2023	-2.76 [-4.45, -1.07]
Xi2023	-2.15 [-3.80, -0.50]
Li2016	-2.69 [-4.44, -0.95]
Wang2021	-1.87 [-3.04, -0.71]
Yue2016	-2.27 [-3.98, -0.56]
Liang2005	-2.63 [-4.44, -0.83]

Supplementary Table 4 Sensitivity analysis of CD4/CD8

**Study**	**MD[95%CI]**
Chen2012	0.30 [0.15, 0.45]
Cui2001	0.28 [0.13, 0.43]
Li2014	0.29 [0.13, 0.44]
Cai2008	0.27 [0.12, 0.41]
Chen2013	0.31 [0.15, 0.46]
Huang2021	0.28 [0.13, 0.44]
Wang2023	0.30 [0.14, 0.45]
Wu2021	0.30 [0.14, 0.46]
Li2016	0.32 [0.20, 0.44]
Wang2021	0.32 [0.16, 0.47]
Yue2016	0.27 [0.13, 0.42]
Liang2005	0.25 [0.11, 0.39]

Supplementary Table 5 Sensitivity analysis of IgA

Study	MD[95%CI]
Cai2008	0.51 [0.07, 0.96]
Chen2013	0.44 [-0.02, 0.90]
Li2016	0.55 [0.07, 1.03]
Liang2005	0.25 [-0.04, 0.55]
Wang2021	0.51 [-0.06, 1.07]
Xi2023	0.40 [-0.08, 0.88]

Supplementary Table 6 Sensitivity analysis of IgG

**Study**	**MD[95%CI]**
Cai2008	1.60 [0.34, 2.85]
Chen2013	1.82 [0.48, 3.17]
Li2016	2.62 [1.03, 4.22]
Liang2005	1.49 [0.31, 2.68]
Wang2021	2.36 [0.05, 4.68]

Supplementary Table 7 Sensitivity analysis of IgM

**Study**	**MD[95%CI]**
Cai2008	0.37 [0.10, 0.65]
Chen2013	0.34 [0.07, 0.62]
Li2016	0.43 [0.15, 0.71]
Liang2005	0.32 [0.03, 0.60]
Wang2021	0.39 [0.01, 0.76]
Xi2023	0.25 [0.05, 0.44]

Supplementary Table 8 Sensitivity analysis of ALB

**Study**	**MD[95%CI]**
Chen2012	5.09 [2.32, 7.87]
Chen2013	4.98 [1.66, 8.30]
Huang2021	4.94 [1.42, 8.47]
Wang2023	4.60 [1.73, 7.46]
Wu2021	5.00 [1.35, 8.65]
Xi2023	2.56 [1.99, 3.14]
